# Effect of Pro-kin visual feedback balance training system on gait stability in patients with cerebral small vessel disease

**DOI:** 10.1097/MD.0000000000014503

**Published:** 2019-02-15

**Authors:** WeiJing Zhao, Hong You, Shangrong Jiang, Hongxia Zhang, Yanling Yang, Min Zhang

**Affiliations:** a Sino-French Department of Neurological Rehabilitation, Gansu Provincial Hospital, Lanzhou; b Department of Neurological, Dunhuang Hospital, Dunhuang, Gansu, China.

**Keywords:** balance, cerebral small vessel disease (CSVD), gait, pro-kin visual feedback training system

## Abstract

Due to the indistinct nature of symptoms for Cerebral Small Vessel Disease (CSVD), diagnosis is often missed. With significant deterioration of movement disorder, risk of falls increases dramatically.

Comparison study was conducted to explore the association between balance function and gait instability, and the treatment effectiveness of visual feedback balance training on the gait disorder in CSVD patients.

Fifty-six patients with CSVD were studied. They were randomly divided into observation and control groups, and were given conventional gait rehabilitation training, including single-leg weight, shift of the center of gravity, step and hip extension training, stepping up and down on stairs with the affected leg, standing up with hip extension and support of the leg, lateral walking, and in situ walking. Training was performed twice a day for 20 minutes each for 4 consecutive weeks. In addition, the observation group received balance training using Pro-Kin visual feedback balance training system. Both groups were evaluated prior and post-treatment using the Tinetti Scale and the Pro-Kin Visual Feedback Balance Training System. For the Tinetti Scale, lower score indicates more serious gait balance dysfunction. For the Pro-Kin, greater length means poorer balance function. Larger area means poorer balance function. Smaller value of the 2 parameters indicates better balance function.

After training, the trajectory lengths and areas of orbital motions were significantly decreased (*P* < .05). However greater decrease was seen in the observation group (*P* < .01). The trajectory length and area for both groups were less when the eyes open than closed (*P* < .01). The Tinetti scores for balance and gait functions of both groups improved significantly (*P* < .05). However, the observation group showed even greater results than the control group (*P* < .01). Results from Person test showed there was a significant correlation between balance and gait functions.

Combination of visual feedback balance training with conventional rehabilitation treatment could gain a greater result than conventional rehabilitation alone. It indicates that balance training may serve as an additional method for gait stability training for CSVD patients.

## 1 Introduction

Cerebral small vessel disease (CSVD) is a series of diseases that could affect small arteries, arterioles, capillaries, venules, and venules in the brain. It causes lacunar infarction, cerebral hemorrhage, subcortical white matter lesions, cerebral microbleeds, and microinfarction.^[1]^ Incidence of cerebral vascular disease has been increasing in recent years. Disruption of the integrity of spinal cord motor system, cortex and basal ganglia fiber connections caused by white matter lesions (WMLs) and lacunar infarction (LI) result in imbalance between the cone system and the extrapyramidal system in CSVD patients. These patients often show a certain degree of gait instability.^[2]^

Before, we have published an article^[3]^ that aims to study the effect of visual balance feedback system on the balance of patients with white matter disease. At the same time, we found that a large proportion of patients with clinical balance disorders originated from CSVD, and most of them were characterized by gait instability, so we expanded the scope of our study for patients with gait instability CSVD (including lacunar infarction, cerebral hemorrhage, subcortical white matter lesions, cerebral microbleeds, and microinfarction). Compared with the articles published in your journal in 2017, the difference is that the scope of the study was expanded, we are not limited to just studying the balance, but also discussion of balance and gait correlation.

In this study, CSVD patients were randomly divided into 2 groups. The control group received conventional gait rehabilitation therapy while the observation group received additional visual feedback balance training. Comparison between these 2 groups was performed to explore the association between the balance function and gait instability, and the effectiveness of visual feedback balance training on the gait disorders in CSVD patients.

## 2 Study subjects and methods

### 2.1 Study participants

Study subjects were 56 patients with CSVD, who were admitted to the Sino-French Department of Neurological Rehabilitation of Gansu Province Hospital between June 2014 and March 2018 (we received informed consent from participants of written consent), The Gansu Province Hospital Application for project ethical approval to carry out the study within its facilities (Ethical Application Ref: 2014-072)

Patient selection criteria are: 45 to 75 years old, suffering from CSVD for more than 3 years, with the presence of LI, WMLs, perivascular space enlargement, cerebral microbleeds, or brain atrophy demonstrated by head magnetic resonance imaging (MRI) findings,^[4]^ Tinetti score <24 points,^[5,6]^ voluntarily receiving assessment and treatment, able to understand the instructions from the therapist or the machine, and able to complete all the required action.

Patient exclusion criteria are: showing cognitive, visual, or corporative difficulties, showing possible presence of cardiogenic embolism and cerebral infarction of large area, showing high white matter signals caused by other diseases such as multiple sclerosis, metabolism encephalopathy, toxic encephalopathy, or intracranial multiple vasculitis, showing potential evidence of other neurological diseases, showing evidence of severe osteoarthropathy and severe joint and muscle pain which could constrain the patient movement.

The qualified patients were randomly (Randomization sequence was created using Medsci Randomization tool) divided into an observation group and a control group, with 28 patients each. In the observation group, there were 16 males and 12 females, with average age of 61.18 ± 8.79 years old, and average duration of disease of 2.07 ± 0.86 years. There were 8 cases with acute lacunar infarction diagnosed based on cranial MRI, 8 cases with white matter lesions, 6 cases with cerebral microbleeds, 4 cases with amplification of perivascular spaces, and 2 cases with brain atrophy. In the control group, there were 13 males and 15 females, with average age of 65.39 ± 8.76 years old, and average duration of the disease of 2.25 ± 0.75 years. There were 7 cases of acute lacunar infarction diagnosed based on cranial MRI, 10 cases of white matter lesions, 4 cases of brain microbleeds, 3 cases with amplification of perivascular spaces, and 4 cases with brain atrophy. More detailed information, which includes gender, age, weight, case type etc., is listed in Table 1. These 2 groups do not show statistical difference, and hence data from these 2 groups are statistically comparable.

**Table 1:**
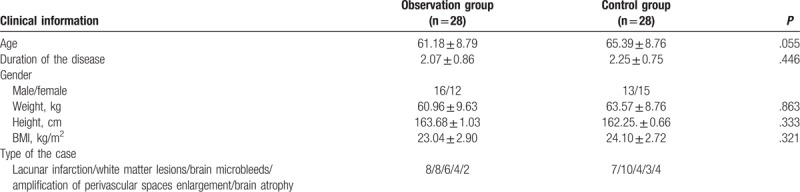
General information of the selected patient.

### 2.2 Methods

#### 2.2.1 Training methods

**Figure 1:**
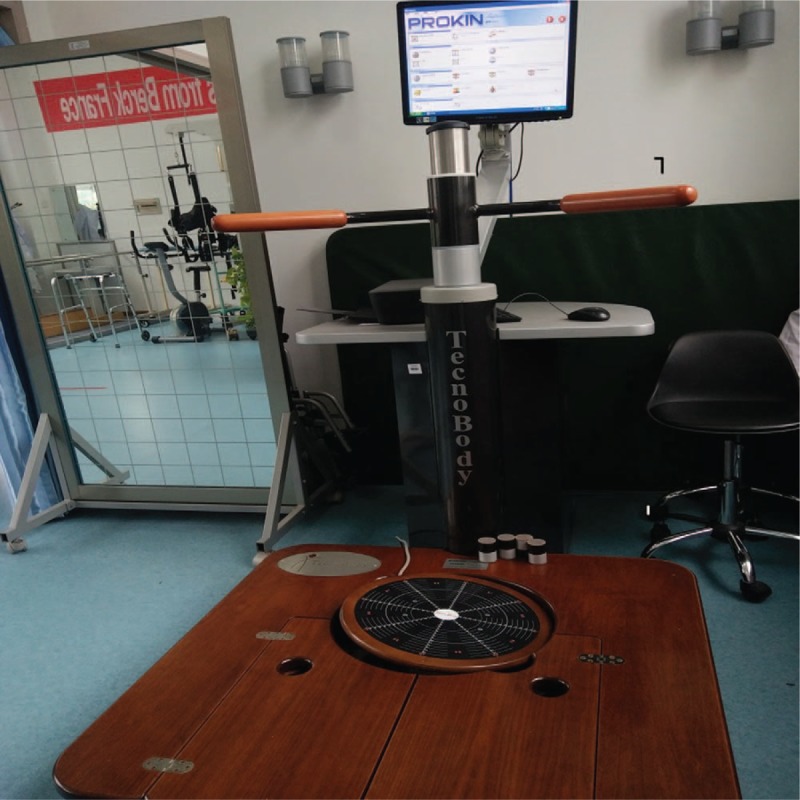
Pro-kin visual feedback balance training system.

All the patients were given conventional gait rehabilitation training, including single-leg weight, shift of the center of gravity, step and hip extension training, going up and down stairs with the affected leg, standing up with hip extension and support of the leg, lateral walking, and in situ walking.^[7]^ Training was conducted twice a day for 4 consecutive weeks. Each training session lasted 20 minutes. The observation team received additional balance training using Pro-Kin visual feedback balance training system (Tecnobody, Model PK254, Italy, Fig. 1). The training module built in the Pro-Kin trainer was utilized to perform static and dynamic balance training. The balance training is shown as follows.

##### 2.2.1.1 Static balance training

**Figure 2:**
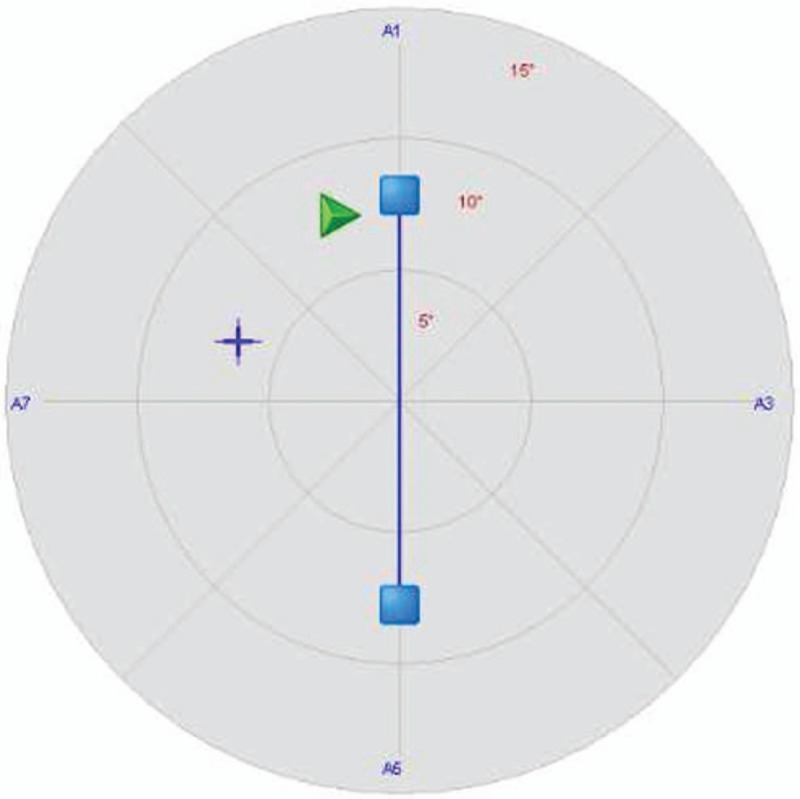
Static balance training interface.

Position the patient in the middle of the balance board with 2 feet forming an angle of 60° (Fig. 2). Then place a fix lock beneath the balance board. The position of the cursor displayed on the monitor indicates the center of gravity of the patient. With the guidance of the rehabilitator, the patient tries to move the position of the cursor on the screen by changing his/her center of gravity.

##### 2.2.1.2 Dynamic balance training

**Figure 3:**
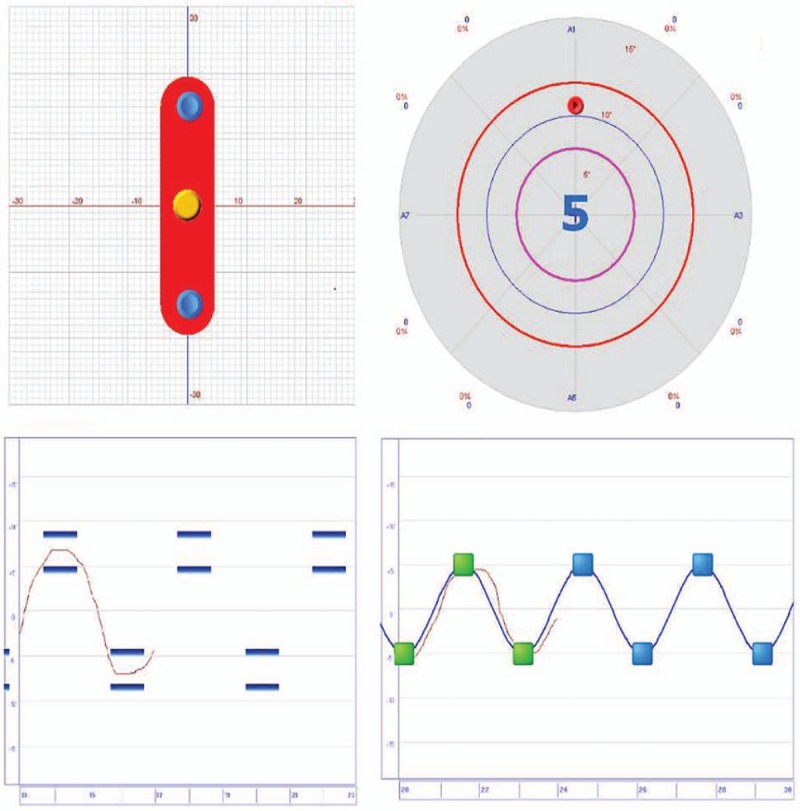
Dynamic balance training interface.

Remove the fix lock beneath the balance board (Fig. 3). Position the patient with single foot on the balance board and the other foot on the ground. The built-in modules “multijoint,” “equilibrium,” and “tricks” are selected. The patient tries to move the cursor on screen by using lower limb joints only.

The training was carried out twice a day, 15 minutes each session, with difficulty level gradually increased.

#### 2.2.2 Evaluation methods

Both groups were evaluated before and after treatment using Tinetti scale^[8]^ and Pro-Kin Visual Feedback Balance Training System.^[9]^ Tinetti scale includes balance and gait testing with the full score of 28 points. There are 9 testing items with 16 points on balance test, and 8 items with 12 points on gait test. A patient with Tinetti scale scored less than 24 points indicates balance dysfunction, scored less than 15 points indicates danger of falling. The lower the score a patient gains, the more the patient suffers from gait balance dysfunction. For all the patients, the static stability module of the Pro-Kin visual feedback balance training system was used to obtain the relevant parameters of the center of pressure (COP), which include length and area of the movement trajectory. The length of the trajectory refers to the length of the trajectory of the pressure center. The greater the length is, the poorer the balance function the patient shows. The area of the trajectory refers to the area surrounded by the trajectory of the center of the body pressure. The larger the area is, the poorer the balance function the patient demonstrates. The smaller the value of the 2 parameters is, the better the balance function the patient enjoys.

#### 2.2.3 Statistical methods

Spss20.0 statistical software was used for analysis. Results were expressed in the format of x ± s. *t* tests were used for intergroup comparison, and *χ*^2^ test was used for comparison of quantitative data. *P* < .05 was considered to be statistically different.

## 3 Results

### 3.1 General information

(Table 1: General information of the selected patients) The general information between the 2 groups was not statistically different (*P* > .05).

### 3.2 Comparison of the changes

After training (Table 2), the trajectory lengths and trajectory areas of orbital motions were significantly decreased (*P* *<* *.05*) in both groups. This holds true for both cases where patents keeping eyes open and closed. However, the decrease in the observation group was more profound (*P* < .01). The trajectory length and area were less when the patients keeping eyes open than closed for both groups (*P* *<* *.01*)

**Table 2:**
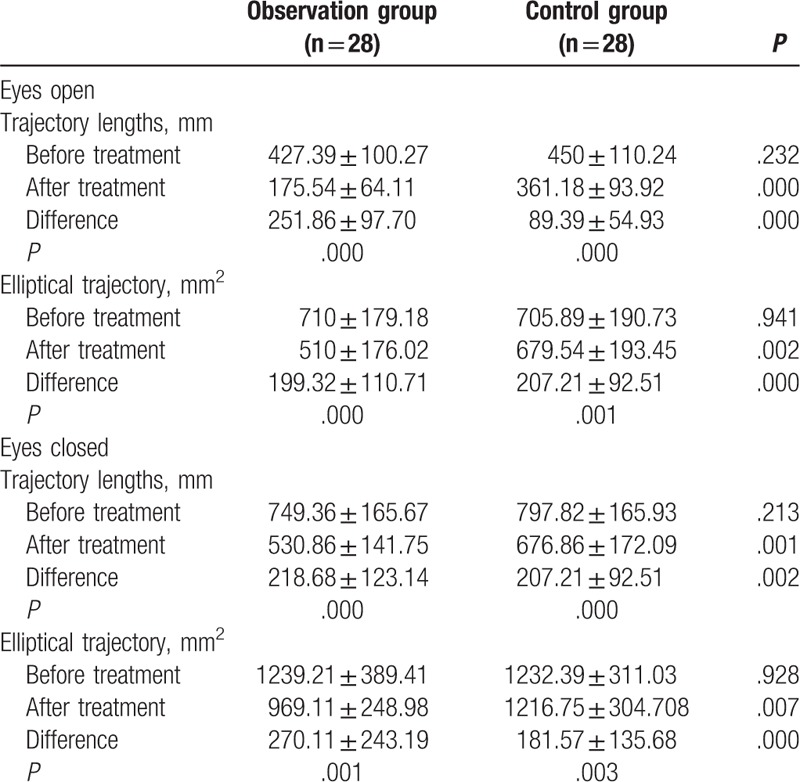
Comparison of the length for eyes closed and open and the elliptical trajectory between the 2 groups before and after training.

After training (Table 3), the Tinetti scores were improved significantly after training for patients’ balance and gait function for both groups (*P* *<* *.05*). However, the observation group showed significantly greater increase than the control group (*P* *<* *.01*). Person test was applied to evaluate the correlation between the gait and balance functions. It is found that these 2 functions are positively correlated, with the bilateral interval confidence level 0.01.

**Table 3:**
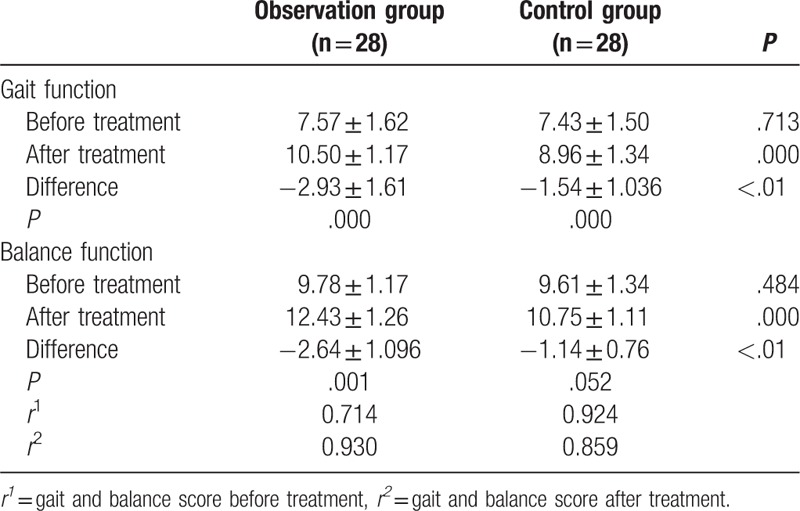
Comparison of the Tinetti score of the 2 groups before and after training.

## 4 Discussions

The interference effects of Pro-kin visual feedback system on stroke patients with hemiplegia after stroke and elderly adults at risk of falling have been reported. For instance, Xiaoxia et al^[10]^ used Pro-Kin balance training system to improve balance and reduce falls risk in stroke patients, and showed that Balance training can improve the balance ability in stroke patients, and reduce the risk of fall. The Pro-Kin balance training is more effective. It has been reported^[11]^ that PRO-KIN balance instrument shows high validity, reliability, and sensitivity for the prediction of falls in old people. In these studies, gait and balance disorders are often appeared as the clinical manifestations of the study subjects. It has been reported that CSVD accounts for 25% to 50% of stroke,^[12]^ increasing the risk of stroke by 1-fold.^[13]^ Due to the hardly noticeable symptoms of CSVD, diagnosis is often missed. Slow movement as the symptom of early phase CSVD is usually neglected as it is often taken as the normal sign of aging. The risk of falls worsens dramatically when movement disorder significantly deteriorates. To the best of our knowledge, this is the first study that applies Pro-kin visual feedback system to intervene the balance and gait of CSVD patients.

Movement disorder, found in 35% of patients with CSVD,^[14]^ can be found in 2 forms: gait disorder and balance disorder. Due to the insignificant impact of the early stage gait dysfunction on overall life quality in CSVD patients, patients often oversee it for a long time. However, early intervention has profound impact on preventing falls and keeping life quality from deterioration. Normal walking is a complex process that requires continuous and instantaneous dynamic balance, which constantly destroys and rebuilds balance. As Bobath in 1978 recommended: “In order to prepare for a normal walk, first of all balance should be practiced, support standing and shifting.” Berg balance scale can only be used to evaluate the balance function, while Tinetti assessment tool consists of multiple tests for both gait and balance assessments, and can manifest the correlation between the 2 scales. Hence, instead of Berg balance scale, Tinetti assessment tool was used in this study. The Tinetti score of patients in control group after treatment was increased by 16% compared with that before treatment, in which the balance and gait scores increased by 19% and 21%, respectively. Besides, the Tinetti score of patients in observation group after treatment increased by 32% compared with that before treatment, in which the balance and gait scores increased by 27% and 39%, respectively. Altogether, Tinetti scores were increased in both groups after treatment, but the increase was more significant in observation group compared with control group, with higher scores for both gait and balance assessments. Comparison of the gait and balance scores before and after training for both groups using Person test showed the balance and gait function are closely correlated both prior to and post training with the bilateral confidence interval as 0.01. For CSVD patients with occult gait disorder symptoms, clinical symptom is usually not gait abnormalities but decline in balance function. Therefore, balance function should be assessed for early detection of the hidden dangers of dyskinesia during the diagnosis of CSVD. During assessment of patient motor function,^[15]^ Tinetti score should be used in addition to the Berg score to identify the correlation between gait and balance function. For patients receiving training in gait function, balance training should also be considered.

The study by Lixia Zong and Kun Jiang,^[16]^ which analyzed the gait characteristics of patients with cerebral vascular disease, showed that more than half of patients exhibit gait dysfunction. Main symptoms include walking speed slowing down, mopping steps, step with wide base, and varied bilateral steps. However, their study has only analyzed the presence of gait features in patients with cerebrovascular disease, but the reason underlying these gait features is not their main focus. In the present study, by using Pro-kin visual feedback balance training system, we compared the length of motion trajectory and the area of motion ellipse was compared in CSVD patients before and after treatment. The lower the motion trajectory length and the motion ellipse area, the better the balance function in patients with CSVD. Therefore, we suggest that the loss of balance function may be the reason for slower gait speed, shorter step length, wider base of support, and greater bilateral step asymmetry among CSVD patients.

Some studies^[17]^ have shown that visual system and proprioception could affect gait and balance independently and interactively, with the impact of the visual system greater than that of proprioception on gait and balance. Control of body balance relies on visual input, proprioception, and input from the vestibular system.^[11]^ When the sense of balance is interfered, the neck muscles contract to keep the head upright and the sight horizontal to restore the original upright body position and then achieve balance.^[18]^ This study demonstrated that, in control group, the lengths of motion trajectory were decreased by 20% (eyes open) and 26% (eyes closed) after treatment compared with those before treatment, whereas the areas of motion ellipse were reduced by 29% (eyes open) and 15% (eyes closed) after treatment compared with those before treatment. Likewise, in observation group, the lengths of motion trajectory were decreased by 59% (eyes open) and 29% (eyes closed) after treatment compared with those before treatment, whereas the areas of motion ellipse were reduced by 28% (eyes open) and 28% (eyes closed) after treatment compared with those before treatment. This study showed that the length of the motion trajectory and the area of the motion ellipse are smaller with eyes open than closed in both groups prior and post treatment. But the observation group achieved better results with eyes open after treatment. Pro-kin training system utilizes visual feedback to train body balance function. Patients dynamically react to constant swinging movements of the balance board. This process produces various stimuli to the brain center, to recover damaged nervous system and coordination functions. These will improve overall walking stability. More importantly, Pro-kin training system can remove continuous visual feedback. It enables the comparison of the sensory level of physical and metal motor sensation generated throughout the exercises. It can ultimately lead to the reestablishment of the correct depiction of proprioceptive and muscular motor sensation.

The improvement of balance and gait in CSVD patients by Pro-Kin balance training system is superior to the traditional balance and gait training methods, which may be attributed to the unique characteristics of Pro-Kin balance system. This system contains different user-friendly modes, such as force platform, the evaluation of balance, and training mode, which may attract patients to participate into the training and accelerate the recovery of their balance dysfunction.

Although this clinical research has reached its aim, there were some unavoidable limitations, including small sample size, no grading of leukoencephalopathy, and no localization of lacunar infarction and cerebral microhemorrhage. Moreover, this study analyzed the changes in Tinetti score, motion trajectory lengths, and motion ellipse areas before and after training, but the neural network interactions in the related brain regions have yet to be investigated. Thus, these aspects warrant further investigation.

## 5 Conclusions

Combination of visual feedback balance training with conventional rehabilitation treatment could gain a greater result than conventional rehabilitation alone. It indicates that balance training may serve as an additional method for gait stability training for CSVD patients.

## Acknowledgments

The authors thank all of the study participants.

## Author contributions

**Conceptualization:** Weijing Zhao, Hong You, Hongxia Zhang, Yanling Yang.

**Data curation:** Weijing Zhao, Hong You, Hongxia Zhang, Yanling Yang.

**Formal analysis:** Weijing Zhao, Hong You.

**Funding acquisition:** Hong You.

**Investigation:** Weijing Zhao, Shangrong Jiang, Hongxia Zhang.

**Methodology:** Weijing Zhao, Shangrong Jiang, Hongxia Zhang.

**Project administration:** Weijing Zhao, Shangrong Jiang.

**Resources:** Weijing Zhao, Shangrong Jiang, Min Zhang.

**Software:** Weijing Zhao, Hong You, Shangrong Jiang, Hongxia Zhang, Yanling Yang, Min Zhang.

**Supervision:** Weijing Zhao, Hong You, Yanling Yang, Min Zhang.

**Validation:** Weijing Zhao, Hong You, Yanling Yang, Min Zhang.

**Visualization:** Weijing Zhao, Min Zhang.

**Writing – original draft:** Weijing Zhao.

**Writing – review & editing:** Weijing Zhao, Hong You, Shangrong Jiang.

Weijing Zhao orcid: 0000-0002-7163-0865.
